# Differential Surface Interactions and Surface Templating of Nucleotides (dGMP, dCMP, dAMP, and dTMP) on Oxide Particle Surfaces

**DOI:** 10.1021/acs.langmuir.2c01604

**Published:** 2022-11-29

**Authors:** Izaac Sit, Eleanor Quirk, Eshani Hettiarachchi, Vicki H. Grassian

**Affiliations:** ^†^Department of Nanoengineering and ^‡^Department of Chemistry & Biochemistry, University of California San Diego, La Jolla, California 92093, United States

## Abstract

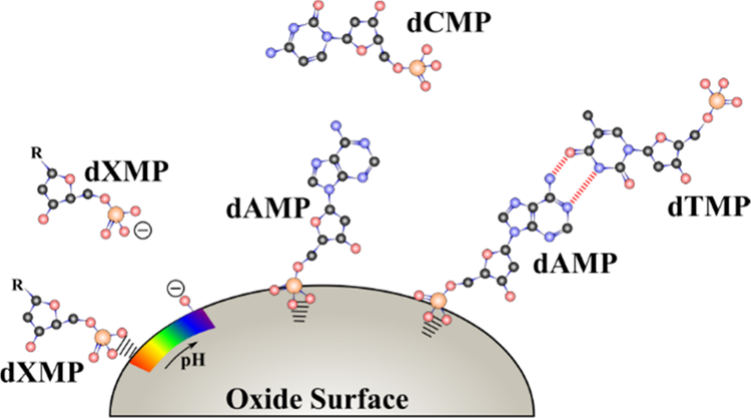

The fate of biomolecules in the environment depends in part on understanding the surface chemistry occurring at the biological–geochemical (bio–geo) interface. Little is known about how environmental DNA (eDNA) or smaller components, like nucleotides and oligonucleotides, persist in aquatic environments and the role of surface interactions. This study aims to probe surface interactions and adsorption behavior of nucleotides on oxide surfaces. We have investigated the interactions of individual nucleotides (dGMP, dCMP, dAMP, and dTMP) on TiO_2_ particle surfaces as a function of pH and in the presence of complementary and noncomplementary base pairs. Using attenuated total reflectance-Fourier transform infrared spectroscopy, there is an increased number of adsorbed nucleotides at lower pH with a preferential interaction of the phosphate group with the oxide surface. Additionally, differential adsorption behavior is seen where purine nucleotides are preferentially adsorbed, with higher surface saturation coverage, over their pyrimidine derivatives. These differences may be a result of intermolecular interactions between coadsorbed nucleotides. When the TiO_2_ surface was exposed to two-component solutions of nucleotides, there was preferential adsorption of dGMP compared to dCMP and dTMP, and dAMP compared to dTMP and dCMP. Complementary nucleotide base pairs showed hydrogen-bond interactions between a strongly adsorbed purine nucleotide layer and a weaker interacting hydrogen-bonded pyrimidine second layer. Noncomplementary base pairs did not form a second layer. These results highlight several important findings: (i) there is differential adsorption of nucleotides; (ii) complementary coadsorbed nucleotides show base pairing with a second layer, and the stability depends on the strength of the hydrogen bonding interactions and; (iii) the first layer coverage strongly depends on pH. Overall, the importance of surface interactions in the adsorption of nucleotides and the templating of specific interactions between nucleotides are discussed.

## Introduction

Aqueous environments in groundwater are a complex milieu comprised of oxyanions, biomolecules, and heavy metals, just to name a few components. These interact with each other and geochemical surfaces present in the environment.^1−7^ For biomolecules, interactions with mineral particle surfaces, especially on high surface area nanoscale sized particles that act as excellent adsorbents,^1,4,5,8−10^ can change the biomolecular structure, physicochemical properties, and electronic states.^2,6,7,11,12^ DNA, oligonucleotides, and nucleotide components are often found in the environment through cellular lysis, leaky sewage pipes, and active cellular secretion.^13,14^ The role of surfaces and the interactions that occur with surfaces in altering DNA decay rates or stabilization is not fully understood.^15^ The fate of environmental DNA and its persistence in aquatic environments are important questions that remain to be answered.

Titanium dioxide particles are found in the environment as natural minerals or anthropogenic engineered nanomaterials and can provide a surface for biomolecules to adsorb onto.^7,12^ These nucleotides compete with other species that are present in environment aqueous systems that make up the ecological corona surrounding the particle. This ecological corona is dependent on the components present in the environment surrounding the particle as higher affinity molecules displace lower affinity species. However, to study the evolution of an ecological corona in a multicomponent milieu, fundamental interactions need to be understood of single- or two-component studies to build up in complexity and predict the behavior to model realistic environments. Additionally, adsorbed DNA can translocate far distances, where transfer of non-native genetic information can occur.^13,16^ Despite being transported through different biomes and exposed to a milieu with components with varying surface affinities, adsorbed DNA can resist degradation.^15,17,18^ Furthermore, prebiotic life was hypothesized to originate from the adsorption of biomonomers onto surfaces, increasing the local concentration and undergoing polymerization to form biomacromolecular structures, like DNA and proteins.^19,20^ To investigate how DNA is stabilized on surfaces, it is first necessary to understand the surface chemistry with the individual building blocks, nucleotides.

For the reasons noted above, it is important to use molecular-based probes to interrogate the surface chemistry to gain insight into the reversibility or irreversibility of adsorbed nucleotides and specific surface interactions. There have been several studies of nucleotide and nucleoside adsorption onto clay and iron oxide particles but few on titanium dioxide.^1,10,20−25^ The studies thus far on TiO_2_ have focused on quantifying nucleotide surface coverage but did not investigate the details of the surface interactions.^26^ Cleaves et al. investigated adsorbed nucleobases, nucleosides, and monophosphate nucleotides and concluded that monophosphate nucleotides are more strongly bound to rutile surfaces compared to the nucleobases.^21^ Zhang et al. adsorbed oligonucleotides onto TiO_2_ and suggested the adsorption occurred through backbone phosphate groups but did not discuss in detail the coordination of phosphate with the surface.^27^ In another study, Schmidt et al. investigated the adsorption of environmental DNA (eDNA) onto goethite and observed the preferential adsorption of the phosphate backbone to the surface.^28^ Further understanding of the interaction of DNA and the components that make up DNA would require probing the interactions of the phosphate group. The phosphate group binding energies differ between monodentate and bidentate modes, which influence how stable nucleotides and DNA are on surfaces.^3^

In this study, in situ attenuated total reflectance-Fourier transform infrared (ATR-FTIR) spectroscopy was used to probe surface interactions of monophosphate nucleotides with TiO_2_ (anatase) surfaces. Solution-phase spectra were compared to adsorbed spectra as a function of pH to better understand bio–geo interactions and the effects of relevant environmental conditions. To build complexity, two-component nucleotide adsorption showed interesting and different interactions between complementary and noncomplementary base pairs. From these studies, it is shown for the first time that there is differential adsorption of nucleotides and different surface interactions as observed in competitive and complementary base adsorption. Overall, this study provides insight into the bio–geo interactions as well as nucleotide templating that could provide insights into prebiotic DNA interactions.

## Materials and Methods

### Materials

2′-deoxycytidine-5′-monophosphate (dCMP), 2′-deoxyguanosine-5′-monophosphate (dGMP), 2′-deoxyadenosine-5′-monophopshate (dAMP), 2′-deoxythmidine-5′-monophosphate (dTMP), sodium chloride, 1N hydrochloric acid, and 1N sodium hydroxide were purchased from Sigma-Aldrich. Anatase TiO_2_ particles were purchased from US Research Nanomaterials stock number #US3498. All chemicals were used without additional modification or purification. For clarity, nucleotides have three main functional groups, the nitrogenous ring, the ribose sugar ring, and the phosphate group. The nitrogenous ring and ribose are defined as the nucleosides. The nucleoside with the phosphate group is the nucleotide.

### Particle Characterization

The crystalline phase of TiO_2_ was confirmed and determined with X-ray diffraction using an APEX II ultra-diffractometer with Cu Kα radiation at λ = 1.54056 Å. To determine the primary TiO_2_ particle size, an aqueous suspension of 0.05 g/L was sonicated with a probe sonicator for 60 s with 15 s rest over 30 min in a room temperature water bath. Afterward, a 15 μL aliquot was drop-cast onto a formvar/carbon-coated 100 mesh copper grid and dried. The copper grid was imaged using an 80 kV JEOL-1400 Plus transmission electron microscope. Particle sizes were analyzed using ImageJ software for more than 100 particles. For scanning electron microscopy (SEM) images for particle film morphology, 2.5 mg of the particles was sonicated in 700 μL of water for 30 s. Then, fourteen 5 mm x 5 mm silicon wafers were laid on the ATR crystal, and the colloidal suspension was pipetted into the trough. The solution was dried, and a wafer was imaged using a FEI Quanta FEG 250 SEM at 10 kV.

The specific surface area was determined using a Quantachrome Nova 4200e N_2_ adsorption isotherm under liquid nitrogen. Samples were first degassed at 120 °C for 18 h, and a 15-multipoint isotherm was collected between P/P_0_ of 0.05–0.95.

### ζ Potential Using Dynamic Light Scattering (DLS)

An aqueous solution of 2.5 g/L of TiO_2_ particles was sonicated for 30 min. A solution of 200 μM dGMP, 200 μM dCMP, 200 μM dAMP, and 200 μM dTMP was separately prepared. All solutions were titrated to pH 5 and pH 9 using HCl and NaOH. Minimal titrant was used to ensure negligible changes to concentration. Triplicate ζ potential measurements were taken with Malvern Instruments Zetasizer Nano.

### Attenuated Total Reflectance–Fourier Transform Infrared (ATR-FTIR) Spectroscopy

The ATR-FTIR spectroscopy setup has been previously described.^2^ Briefly, ATR-FTIR spectroscopy is based on the total internal reflection of an infrared beam at an interface between an optically dense medium (ATR crystal) and an optically rare medium (sample). The reflection of the incident beam at the interface creates an evanescent wave that propagates into the sample, where absorption of infrared light can occur, decaying exponentially. The ATR accessory was a horizontal flow cell with an amorphous material transmitting IR radiation (AMTIR) crystal. Infrared spectra were collected using a Nicolet iS10 FTIR spectrometer (Thermo Fisher) equipped with a mercury cadmium telluride detector (MCT/A). Spectra were collected at a resolution of 4 cm^–1^ and averaged over 100 scans in the spectral range extending from 750 to 4000 cm^–1^. All ATR-FTIR spectra were collected and background-subtracted using a linear baseline between 900 and 1800 cm^–1^ with OMNIC 9 software. All spectra were taken after purging atmospheric gases for approximately 30 min with zero air. Adsorption spectra were taken every 5 min.

Solution-phase spectra of dGMP, dCMP, dAMP, and dTMP were taken to compare spectral differences when these nucleotides are adsorbed onto TiO_2_. A solution of 2 mM dGMP in 10 mM NaCl was prepared and titrated to pH 5 or pH 9 using HCl and NaOH. The solution was pipetted onto the AMTIR crystal, and a spectrum was taken using a 10 mM NaCl background-titrated to the appropriate pH. The same was done for the other nucleotides to collect solution-phase spectra for pH 5 and pH 9. The addition of a small volume of titrant has negligible effects on the total ionic strength of the solution.

For single-component adsorption, a TiO_2_ particle thin film was prepared by sonicating 2.5 mg of TiO_2_ in 500 μL of Milli-Q water and pipetting the resulting solution onto the AMTIR crystal. The solution was left to dry overnight, leaving a thin TiO_2_ film. A solution of 10 mM NaCl at pH 5 or 9 was flowed over the thin film using a peristaltic pump at ∼1 mL/min to remove loose particles and collect a background spectrum. A solution of 20 μM dGMP in 10 mM NaCl titrated to pH 5 or 9 was prepared and flowed over the thin film for 180 min. Then, a desorption solution of 10 mM NaCl at the corresponding pH was flowed over the film for 120 min. The same method was done to collect the other three nucleotide single-component adsorption.

For two-component adsorption, a TiO_2_ thin film was prepared, and 10 mM NaCl solution was flowed over to remove loose particles and to collect a background at pH 5 or 9. To keep the number of adsorption sites the same as a single-component system, a solution of 10 μM dGMP and 10 μM dCMP was prepared and titrated to pH 5. The solution was flowed over the thin film for 180 min. Then, a 10 mM NaCl desorption solution was flowed over the film for 120 min. The same method was followed for 10 μM dAMP and 10 μM dTMP, 10 μM dGMP and 10 μM dTMP, and 10 μM dAMP and 10 μM dCMP.

### UV–vis Surface Coverage Quantification

Separate stock solutions of 1 mM each dGMP, dAMP, dCMP, and dTMP were prepared in 10 mM NaCl and titrated to pH 5. A solution of 10 g/L TiO_2_ was prepared in 10 mM NaCl and sonicated for 1 min to create a colloidal suspension. The suspension was titrated to pH 5. Aliquots of the stock solutions were mixed for a final reaction concentration of 5 g/L TiO_2_ and 20 μM nucleotide. These concentrations were analogous to the concentration used in the ATR-FTIR experiments. The reactors were put on a rotator for 2 h, then the reactors were centrifuged, and the supernatants were collected. Fresh 20 μM nucleotide stock solution was added and was reacted for another 2 h. The reactors were centrifuged for a second time, and the supernatants were collected. Both supernatants were analyzed with ultraviolet–visible (UV–vis), and surface coverage was calculated using 20 μM stock measurements and specific surface area measurements. UV–vis was taken with an Agilent Cary 5000 spectrophotometer in the wavelength range of 200–400 nm at a scan speed of 600 nm/min.

## Results and Discussion

### Titanium Dioxide Particle Characterization

**Figure 1:**
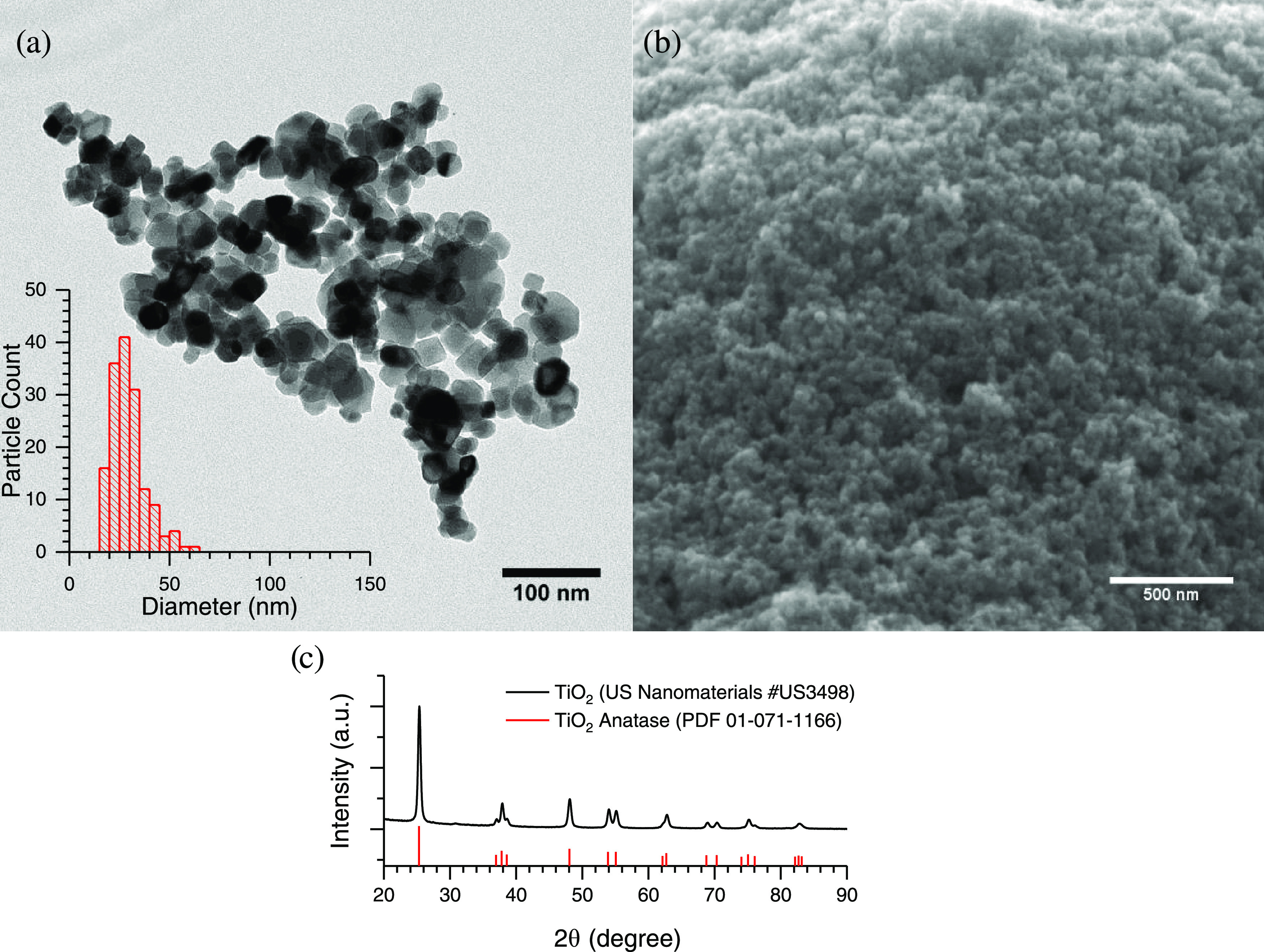
Particle characterization for TiO_2_ particles. (a) TEM micrograph with size distribution analysis (inset); (b) SEM micrograph of a particle thin film; and (c) XRD data of anatase TiO_2_.

The titanium dioxide average particle size was determined to be 29.4 ± 8.5 nm with TEM (Figure 1a). A micrograph of the particle’s thin film can be seen to be porous (Figure 1b). The particles were confirmed to be single-phase anatase with XRD (Figure 1c). The specific surface area of the anatase particles was measured to be 41.4 ± 4.0 m^2^/g.

### Analysis of Solution-Phase and Single-Component Adsorption

**Figure 2:**
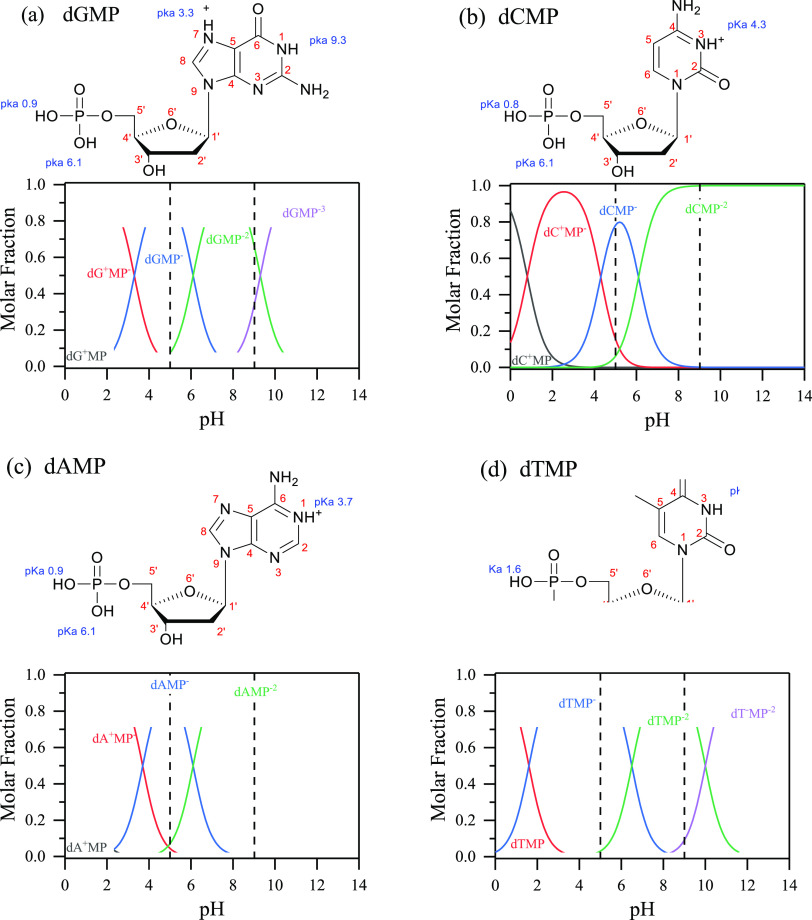
Fully protonated nucleotide structures and their p*K*_a_ values (top) and speciation plots determined from the Henderson–Hasselbalch equation (bottom) are shown for (a) deoxyguanosine monophosphate, dGMP; (b) deoxycytidine monophosphate, dCMP; (c) deoxyadenosine monophosphate, dAMP; and (d) deoxythymidine monophosphate, dTMP.

Figure 2 shows molecular structures of fully protonated forms of dGMP, dCMP, dAMP, and dTMP with p*K*_a_ values of the phosphate groups and nitrogenous rings. The speciation of the four nucleotides at different pHs is shown in Figure 2, and the species present at pH 5 and 9 are shown in Table S1. At pH 5 for dGMP, the amounts of the zwitterionic, monovalent anionic, and divalent anionic forms are 1.8, 91.0, and 7.2%, respectively. For dCMP at pH 5, these percentages change to 15.6, 78.2, and 6.2% for the zwitterionic, monovalent anionic, and divalent anionic forms, respectively. For dAMP at pH 5, these are 4.4, 88.6, and 7.0% for the zwitterionic, monovalent anionic, and divalent anionic, respectively, and for the dTMP, these are 96.9 and 3.1% for the monovalent anionic and divalent anionic, respectively. At higher pH, i.e., pH 9, the phosphate group and nitrogenous rings are fully deprotonated, leading to all nucleotides residing in their divalent or trivalent anionic forms. These speciation forms have been tabulated in Table S1 for all four nucleotides at pH 5 and 9, and other studies show similar calculations.^1,29^ The dominant species for all four nucleotides at pH 5 is monovalent anion, while the dominant species at pH 9 is a divalent anion. Since the nucleotides will be negatively charged at both pH 5 and 9, the surface charge of the TiO_2_ surface will determine the electrostatic surface interactions.

**Figure 3:**
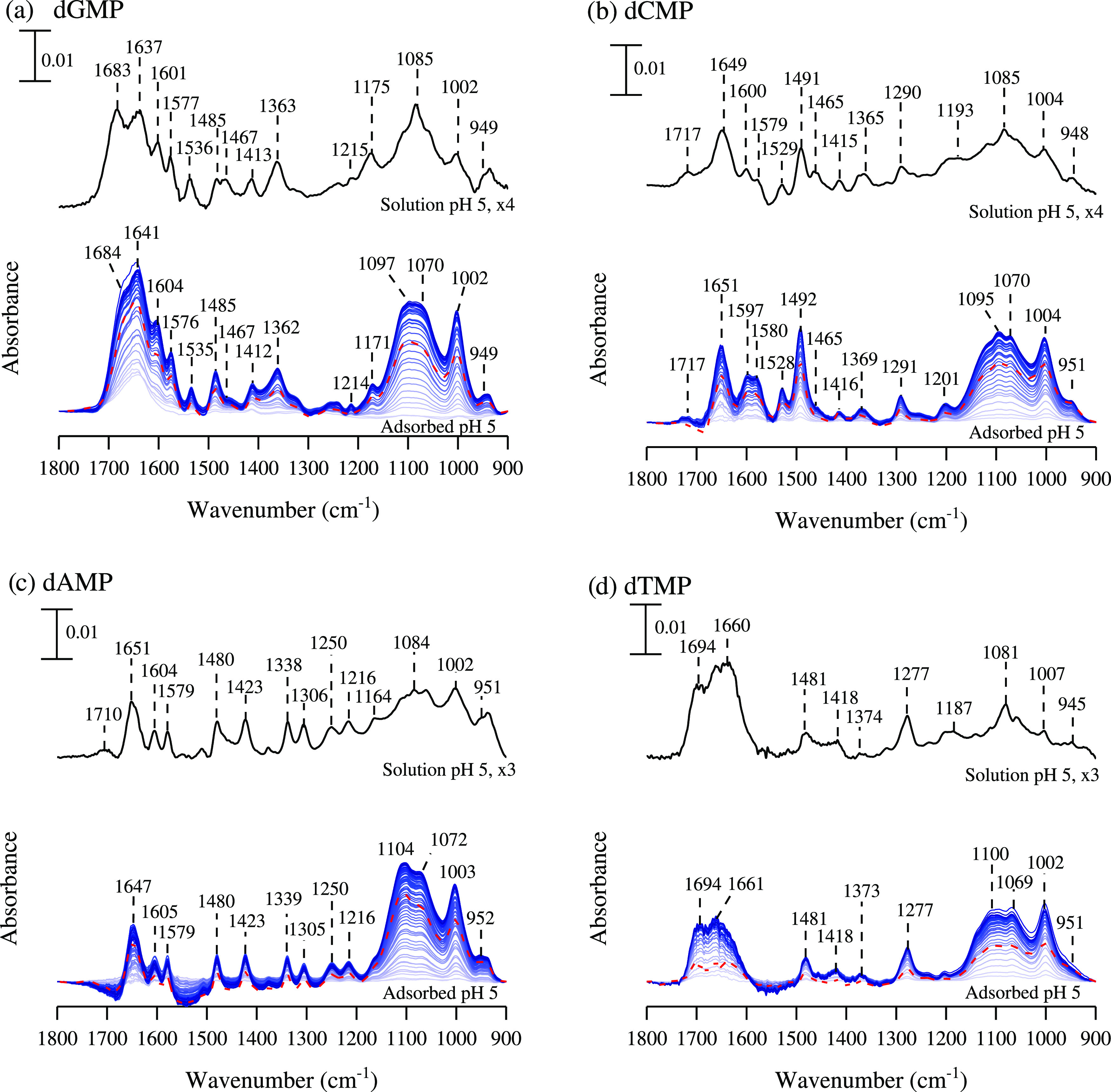
ATR-FTIR spectra at pH 5 of solution-phase nucleotides (top) and adsorbed on TiO_2_ (bottom) for (a) deoxyguanosine monophosphate; (b) deoxycytidine monophosphate; (c) deoxyadenosine monophosphate; and (d) deoxythymidine monophosphate. ATR-FTIR spectra are collected as a function of adsorption time. These spectra are shown every 5 min from light to dark coloration. The red dotted line represents the desorption spectrum after 120 min. Solution-phase spectra have been scaled (3–4x) on the same scale bar.

These speciation forms at pH 5 can be observed in their solution-phase spectra, which are shown in Figure 3 (top), as well as at pH 9, in Figure S1 (top). In general, the region between 1200 to 1800 cm^–1^ can be assigned to the nucleoside while the 900 to 1200 cm^–1^ region can be assigned to the phosphate group. At pH 5, the phosphate band shape for all nucleotides is similar, given that the phosphate protonation state is identical. For all four nucleotides, the main phosphate absorptions can be grouped in the following manner: 1164 to 1093, 1081 to 1085, 1002 to 1007, and 945 to 951 cm^–1^. The 1093 to 1164 and 1081 to 1085 peaks represent the ν_as_(PO_2_^–^) and ν_s_(PO_2_^–^), respectively. The 1002 to 1007 cm^–1^ peak can be assigned to ν(P–O) while the 945–951 cm^–1^ band is assigned to δ(POH). At pH 9, deprotonation occurs, and there is an increase in phosphate symmetry. This leads to a reduction in the number of peaks observed. Mainly, solution-phase nucleotide spectra show three main phosphate absorption bands, a broad 1089 cm^–1^ and two more distinct 934 and 978 cm^–1^ peaks. The broadening of the 1089 cm^–1^ ν_as_(PO_3_^2–^) band paired with the 978 cm^–1^ ν_s_(PO_3_^2–^) when compared to pH 5 is characteristic of a fully deprotonated phosphate group.^5^ The 934 cm^–1^ band δ(POH) is very small because the phosphate speciation is heavily dominated by the doubly deprotonated species. The presence of the phosphate bands at both pH 5 and 9 align well with the nucleotide speciation plot. For the solution-phase nucleoside spectral features in the 1200 to 1800 cm^–1^ region, there are notable differences between pH 5 and 9. The nucleobase for dAMP and dCMP deprotonates, which can be seen by the disappearance of the 1710 and 1717 cm^–1^ δ(NH^+^) bands, respectively. As the dGMP nucleobase undergoes deprotonation from pH 5 to pH 9, the 1693 cm^–1^ δ(NH^+^) band intensity decreases. Vibrational mode assignments for solution and adsorbed nucleotide can be found in Tables S2 and S3.^1,5,22−24,30−32^

Solution-phase nucleotide spectra can be compared to spectra collected of adsorbed phase for nucleotides at pH 5 and 9, where any spectral differences can be attributed to changes due to surface adsorption. Figure 3 (bottom) shows nucleotide adsorption at pH 5, and Figure S1 (bottom) shows nucleotide adsorption at pH 9. Solution-phase spectral intensities are multiplied by a factor of 2–4x and are 100x more concentrated than the bulk solution used for adsorption. This suggests a minimal spectral contribution from the solution phase in the adsorption spectra. For adsorbed nucleotides at pH 5, the nucleoside-related peaks, between 1200 and 1800 cm^–1^, have minimal frequency shifting or broadening as a function of surface coverage. Nucleoside band positions also align with those in the solution phase, suggesting minimal direct interactions of these groups occur with the surface and provide a benchmark to study multilayer interactions. Under desorption conditions, bands only decrease in peak intensities and do not have broadening or frequency changes. This indicates the desorption of weakly bound nucleotides.

When the phosphate absorption spectral region from 900 to 1200 cm^–1^ is overlayed for the solution-phase and adsorbed nucleotide, there is significant broadening due to binding to the TiO_2_ surface (Figure S2). Other studies have reported the preferential adsorption of the phosphate backbone of DNA to surfaces and minimal interaction with the nucleosides.^27,28^ There have been previous studies that show adsorbed oxyanion phosphate and nucleotides contain a mixture of (de)protonated monodentate and bidentate binding modes on metal oxides.^1,3,5,8,25,33^ Interestingly, the adsorbed spectra for all four nucleotides have similar phosphate band shapes between 900 to 1200 cm^–1^. Specifically, there are four major observable bands in the phosphate region, at ca. 951, 1002, 1069, and 1095 cm^–1^. All four bands represent major contributions from phosphate coordination to the TiO_2_ surface for both monodentate and bidentate modes. This suggests that the four singly adsorbed nucleotides have the same binding surface chemistry to the particle surface. Thus, the nucleotides are coordinated to the TiO_2_ surface via the phosphate group.

**Figure 4:**
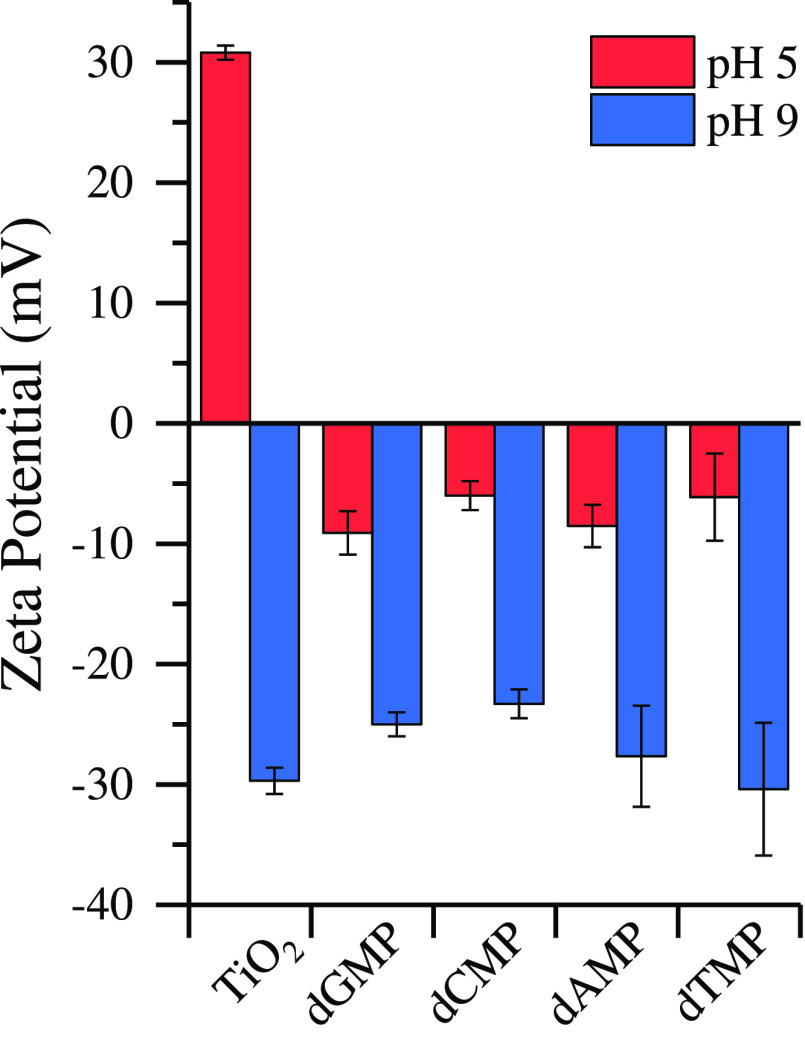
ζ potentials for TiO_2_ and solution-phase nucleotides at pH 5 (red) and pH 9 (blue).

When the pH is increased to 9 (Figure S1), the spectral intensities are drastically attenuated when compared to pH 5, corresponding to a decrease in the number of adsorbed nucleotides. Figure 4 shows the ζ potentials for TiO_2_ and nucleotides at pH 5 and 9. Under acidic conditions, the surface is positively charged, while nucleotides are negatively charged, exhibiting electrostatic attraction. The isoelectric point of TiO_2_ is around 6–6.5.^6,27,34^ Under basic conditions, both the surface and nucleotides are negatively charged, and electrostatic repulsion can occur, reducing the number of adsorbed nucleotides at higher pH values.

**Figure 5:**
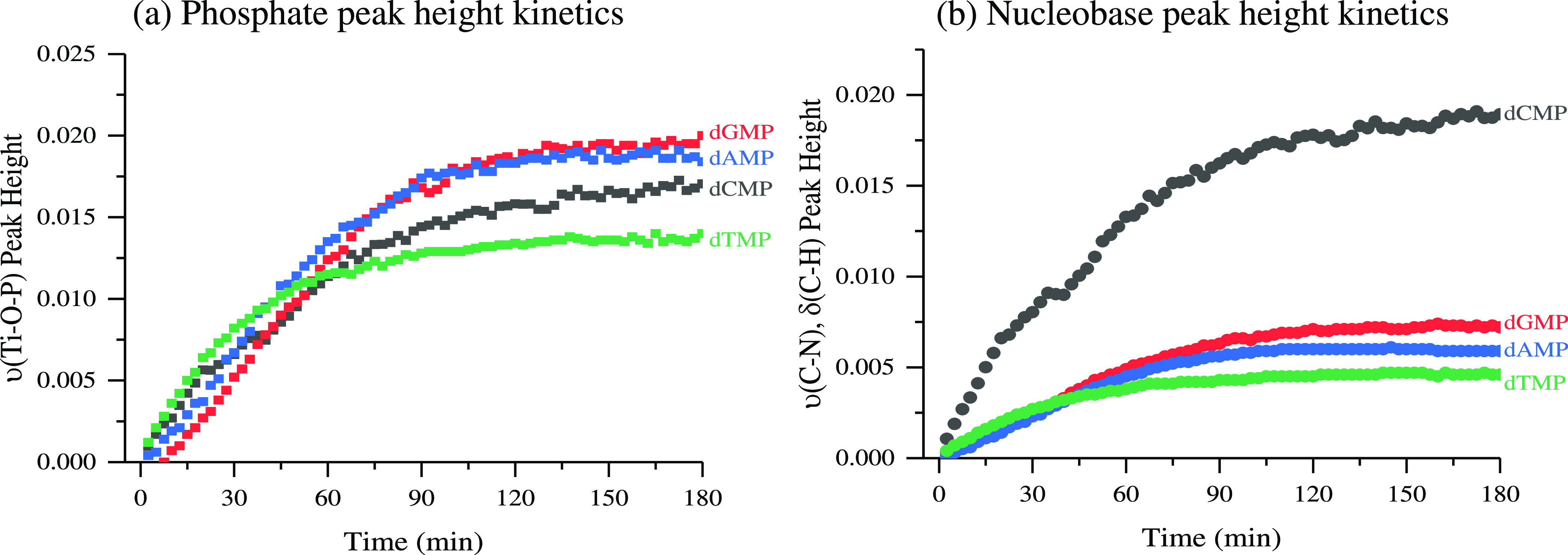
Nucleotide adsorption peak height kinetics onto TiO_2_ at pH 5 using the (a) ∼1000 cm^–1^ ν(Ti–O–P) peak height and (b) ∼1490 cm^–1^ ν(C–N), δ(C–H) vibrational bands.

Figure 5 shows the adsorption kinetics for the ∼1000 cm^–1^ ν(Ti–O–P) and the ∼1490 cm^–1^ ν(C–N), δ(C–H) bands at pH 5 as a function of time, representing the phosphate and nucleoside functional groups, respectively. The peak intensities for both the phosphate and nucleoside functional groups show an exponential increase and a plateau, suggesting the surface has reached the maximum number of adsorbed nucleotides with minimal lateral interactions. This behavior has been previously observed in other studies using peak height kinetics, and it is not surprising to see minimal lateral and absence of multilayer interactions for single-component systems under these conditions.^1,35^ Under desorption conditions, intensities exponentially decrease, eventually slowing the removal of nucleotides. During the desorption stage, the nucleotide intensities do not fall back to zero, suggesting that the nucleotides are irreversibly bound to the surface (Figure S3). This suggests that more weakly bonded nucleotides are removed from the surface, leaving directly coordinated nucleotides on the TiO_2_ surface. Previous studies have shown that the primary binding mode is monodentate but is often dependent on complexing lattice planes, nanoparticle composition, and environmental conditions and is usually a mixture of mono- and bidentate modes.^3,36,37^ These dependencies on various factors lead to differences in adsorption energies that can reversibly desorb loosely H-bound adsorbed species while leaving irreversibly adsorbed monodentate or bidentate complexes.^3^ The irreversibility of adsorbed mono- and polymeric biomolecules are often observed; however, the surface chemistry is often taken for granted and is lightly discussed.^1,2,38−41^ Thus, it is important to understand the fundamental interactions of single or two-component systems with surfaces.

Single-component adsorption constants for pH 5 can be calculated using the exponential portion of the kinetics assuming a first-order adsorption kinetics up to 95% surface coverage. These calculated values are shown in Table 1. The adsorption constants, *k*_ads_, of increasing values follow: dCMP < dTMP < dGMP < dAMP. This suggests that there is a preference of purine nucleotides over pyrimidine nucleotides, and this preferential adsorption to surfaces has been observed in other studies.^37,42−44^ There are several reasons for differential surface affinities observed. First, electrostatic attraction between the more negative purine nucleotides (dGMP and dAMP) over their pyrimidine counterparts (dCMP and dTMP) with the positively charged TiO_2_ surface at pH 5 (Figure 4). The individual ζ potentials for the pair of pyrimidine and purine nucleotides fall within standard deviations; however, it is clear that the purine nucleotides have a lower ζ potential compared to pyrimidine nucleotides. This confirms the preference of purine over pyrimidine nucleotides where electrostatic interactions may play an important role in the interaction with the surface. Second, the purine nucleotides may have a greater van der Waals interaction with the surface compared to pyrimidine nucleotides.^44,45^ Third, G and A nucleobases are less soluble than C and T, favoring surface adsorption of G and A to particle surfaces.^43,45^ Only through a detailed computational study would these different causes to surface affinity address these different interactions.

**Table 1:** Relative First-Order Adsorption Constants to dAMP up to 95% Surface Saturation for Four Nucleotides

	*k*_ads_/*k*_ads,dAMP_
dAMP	1.000
dGMP	0.920
dTMP	0.886
dCMP	0.762

**Figure 6:**
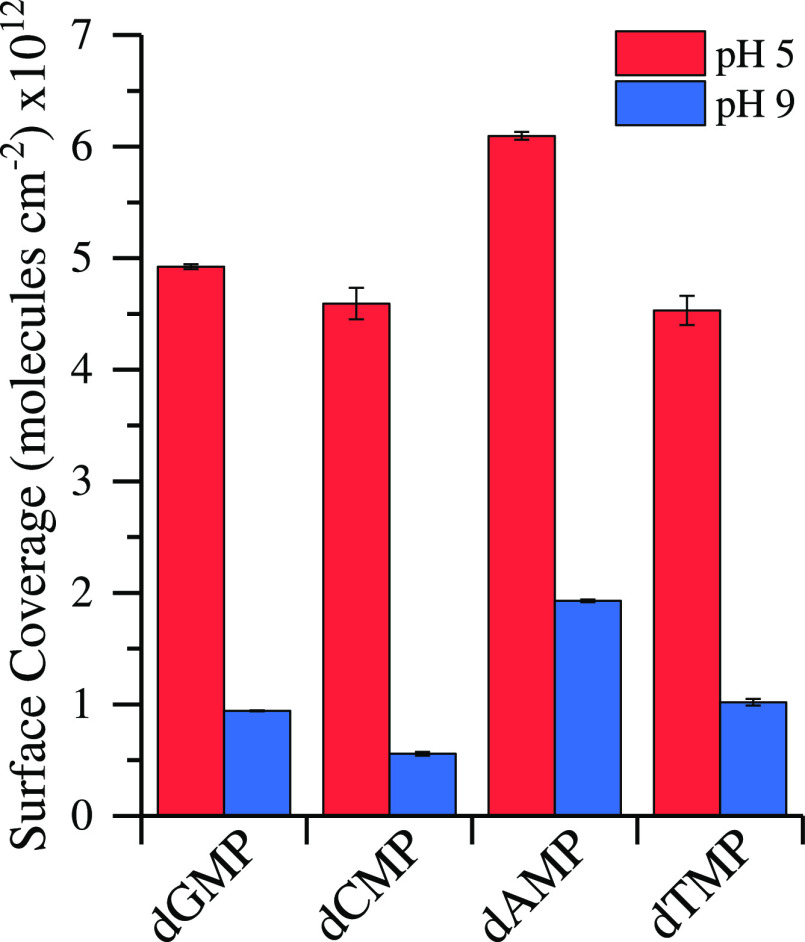
Single-component nucleotide surface coverage on TiO_2_ at pH 5 and 9 in 10 mM NaCl. pH 9 surface coverages were scaled from pH 5 using ratioed ATR-FTIR adsorption intensities.

Figure 6 shows nucleotide surface coverage at pH 5 in increasing values: dTMP, dCMP < dGMP < dAMP. dTMP and dCMP are within the standard deviation and have similar surface coverages. However, the purine nucleotides have a higher surface coverage than pyrimidine nucleotides. These results align well with similar quantitative adsorption studies.^21,36^

### Analysis of Two-Component Noncomplementary and Complementary Base Pair Adsorption

**Figure 7:**
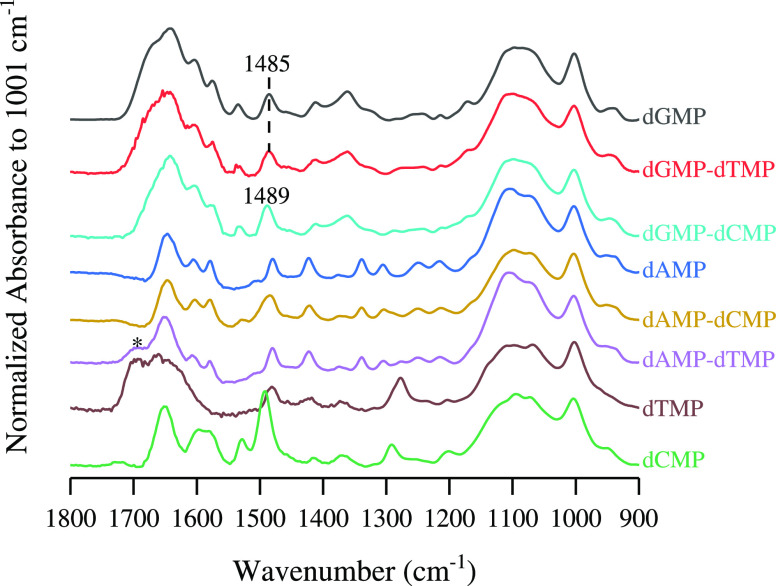
ATR-FTIR spectra at pH 5 of nucleotide adsorption at 180 min on TiO_2_. Single-component adsorption is shown for dGMP (black), dAMP (blue), dTMP (brown), and dCMP (green). Equimolar noncomplementary coadsorption shown for dGMP-dTMP (red) and dAMP-dCMP (gold). Equimolar complementary coadsorption shown for dGMP-dCMP (light blue) and dAMP-dTMP (purple). Spectra have been normalized to 1001 cm^–1^. *Refers to the coadsorbed 1694 cm^–1^ ν(C=O) due to dTMP in second layer.

There is typically a mixture of nucleotides in natural aqueous systems, with various surface affinities competing with the surface and interacting as coadsorbates. To increase complexity to single-component experiments and model more realistic systems, two nucleotides were competitively adsorbed onto the surface. Figure S4 shows ATR-FTIR spectra for the adsorption of an equimolar number of nucleotides consisting of either noncomplementary (dGMP-dTMP and dAMP-dCMP) or complementary base pairs (dGMP-dCMP and dAMP-dTMP) on the TiO_2_ surface. Despite the bulk solutions comprised of an equimolar mixture of purine and pyrimidine nucleotides, the adsorption spectral features are highly similar to single-component purine nucleotide spectra over pyrimidine spectra in all four systems. To emphasize this, Figure 7 shows equilibrated spectra for singly, noncomplementary, and complementary adsorbed nucleotides on the oxide surface. In a two-component system, the spectra resemble that of singly adsorbed purine nucleotides, implying the preferential adsorption over the pyrimidine nucleotides. However, there are small spectral contributions from coadsorbed pyrimidine nucleotides. The singly adsorbed dGMP and noncomplementary dGMP-dTMP spectra show identical spectra with similar 1485 cm^–1^ peak position. However, under complementary base pair conditions of dGMP-dCMP, the 1485 cm^–1^ shifts to 1489 cm^–1^ and has a slightly lower intensity which will be discussed later. It is hypothesized that this is due to a hydrogen-bound second layer. In the noncomplementary dAMP-dCMP system, the 1484 cm^–1^ undergoes some broadening, and a small 1528 cm^–1^ peak appears when compared to the single dAMP spectrum. This is due to small spectral contributions from coadsorbed dCMP; however, the surface composition is still dominated by noninteracting adsorbed dAMP. The coadsorbed dTMP in a second layer for the dAMP-dTMP complementary system is observed, noted by the presence of the 1694 cm^–1^ ν(C=O) peak. After desorption, only 20% of this peak remains on the surface, suggesting reversible adsorption. For all spectra, the 900 to 1200 cm^–1^ phosphate region is identical in shape, suggesting that the composition of monodentate to bidentate surface complexation modes would be similar in ratios, regardless of nucleotide derivative or in a multicomponent system.

The relative number of adsorbed pyrimidine nucleotides in a coadsorbed system was estimated by taking a ratio of intensities for single-component to a two-component system. Pyrimidine peaks were chosen that have minimal overlap with purine peaks, specifically the 1694 cm^–1^ for dTMP and the 1292 cm^–1^ peak for dCMP. Table 2 shows the relative adsorbed amount of purine and pyrimidine nucleotides in a coadsorbed system. Both complementary systems (dGMP-dCMP and dAMP-dTMP) have higher amounts of adsorbed pyrimidine nucleotides than noncomplementary systems (dGMP-dTMP and dAMP-dCMP). This suggests that there is a synergistic effect of complementary nucleotides that increase the relative amounts of surface-adsorbed pyrimidine nucleotides when compared to noncomplementary systems.

**Table 2:** Relative Number of Adsorbed Nucleotides for Noncomplementary and Complementary Base Pairs on the TiO_2_ Surface at pH 5

	relative adsorbed nucleotide (%)	
base pair	purine	pyrimidine
dGMP-dTMP	100	0
dGMP-dCMP	89.5	21.5
dAMP-dCMP	82.5	17.5
dAMP-dTMP	70.3	29.7

**Figure 8:**
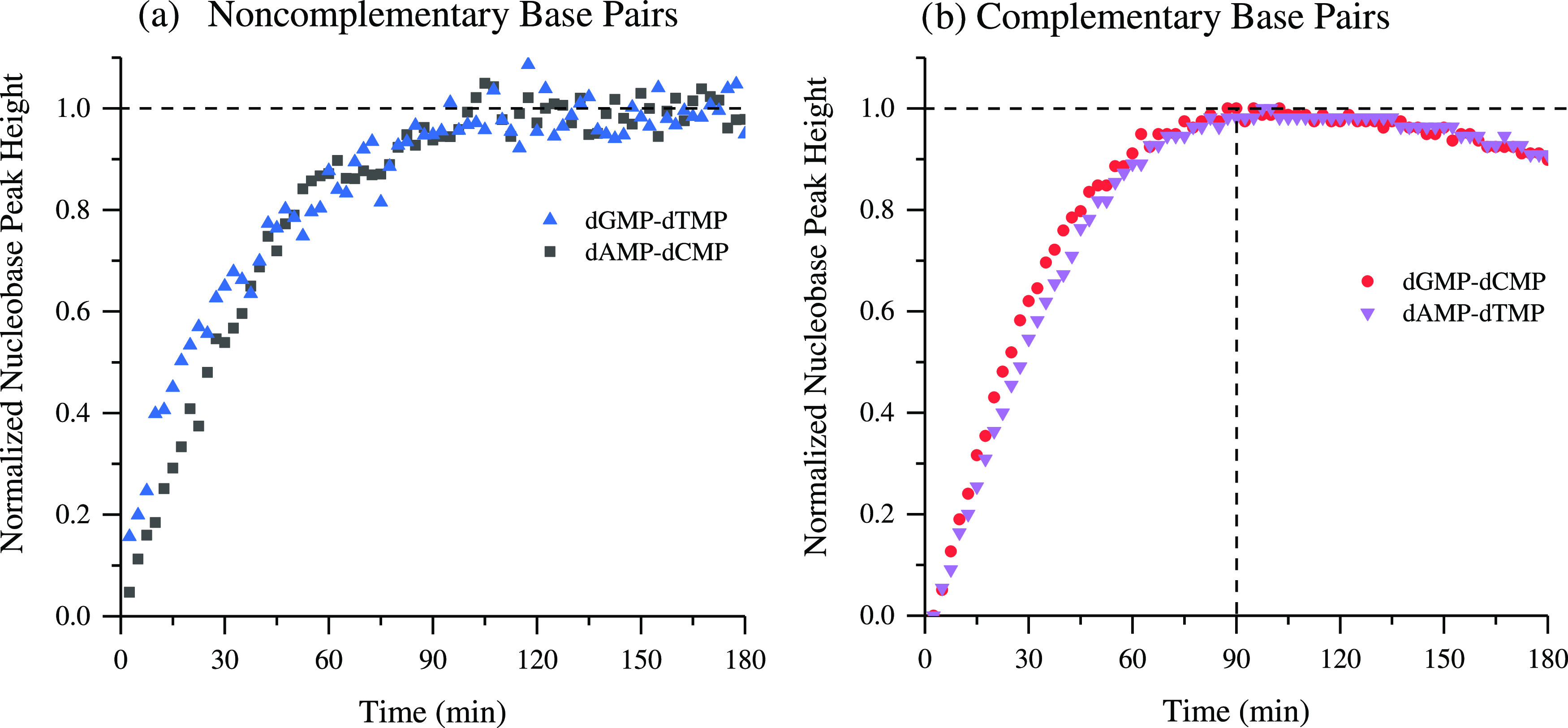
Changes to normalized peak height for two-component nucleotide base pair adsorption onto TiO_2_ at pH 5 using the ca. 1480 cm^–1^ ν(C–N), δ(C–H). (a) Noncomplementary nucleotide base pairs dGMP-dTMP (blue triangle) and dAMP-dCMP (gray square). (b) Complementary nucleotide base pairs dGMP-dCMP (red circle) and dAMP-dTMP (purple down-triangle). The dotted horizontal line emphasizes the decrease in the ν(C–N), δ(C–H) intensity after the 90 min mark for the complementary base pairs (dGMP-dCMP and dAMP-dTMP), where the noncomplementary base pair kinetics plateau (dGMP-dTMP and dAMP-dCMP).

**Figure 9:**
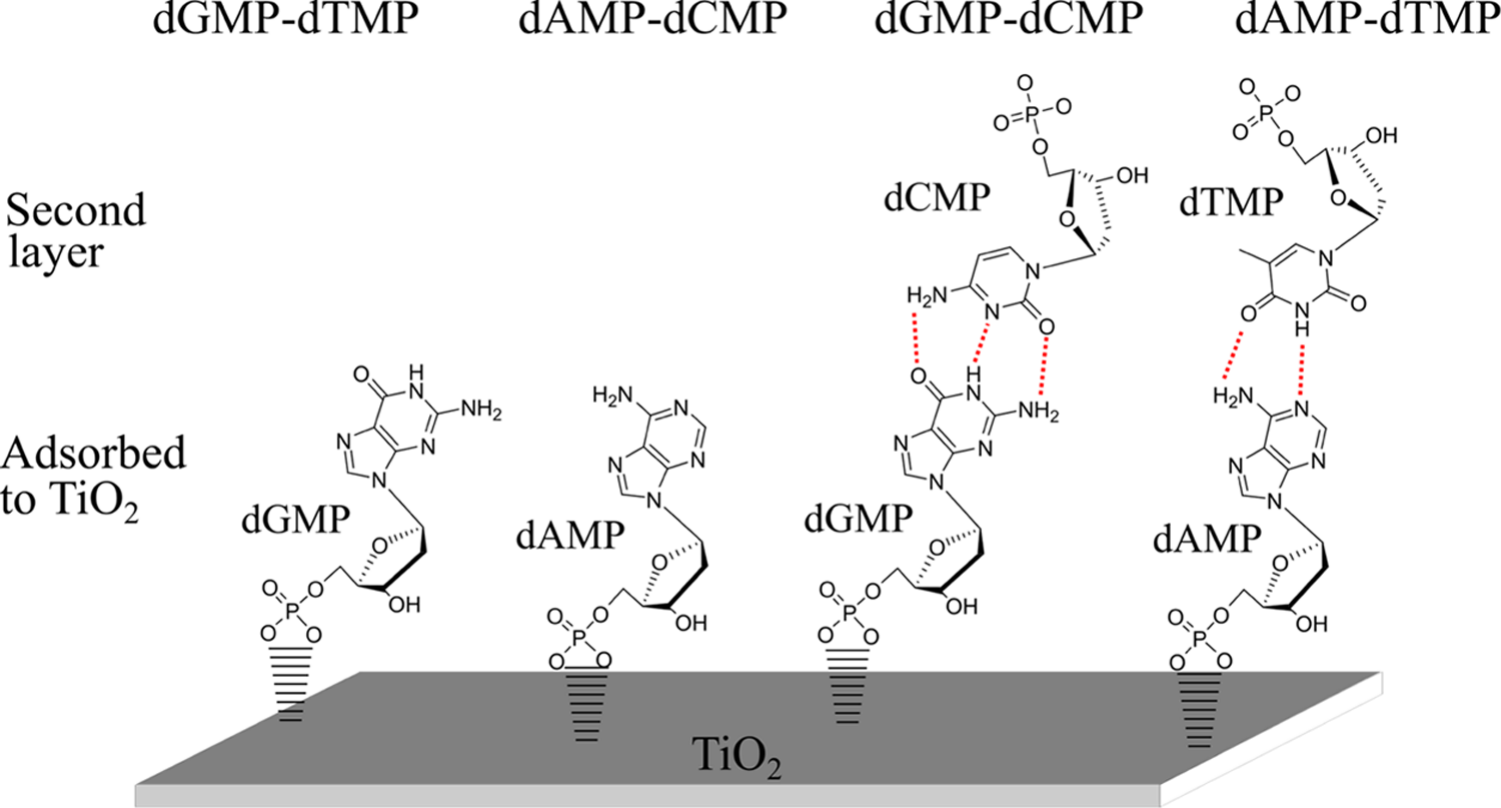
Conceptual representation of two-component adsorption of nucleotide base pairs on TiO_2_. For dGMP-dTMP, there is no interaction between the two nucleotides and dGMP coordinates to the surface while dTMP stays in solution. For dAMP-dCMP, there is no interaction between the two nucleotides, dAMP coordinates to the surface, and dCMP remains in solution. For dGMP-dCMP, there is an interaction between nucleotides with dGMP directly coordinating to the surface while interacting through hydrogen bonds to dCMP in a second layer. For dAMP-dTMP, there is an interaction between nucleotides with dAMP directly coordinating to the surface while interacting through hydrogen bonds to dTMP in a second layer.

The dGMP-dTMP and dAMP-dCMP noncomplementary coadsorption peak height kinetics are shown for the nucleobase (Figure 8a) and phosphate (Figure S5a) functional groups. The kinetics are identical to single-component adsorption such that it is monotonically increases with exponential initial growth and plateaus. The nucleotides are irreversibly bound to the surface as desorption does not fully remove the nucleotides (Figure S6a,b). Figure 9 shows a conceptual representation of the preferential direct coordination of purine (dGMP or dAMP) nucleotides to the TiO_2_ surface without a second layer and free pyrimidine (dTMP or dCMP) nucleotides in bulk. Additionally, the nucleotides have minimal lateral interactions with coadsorbates and do not form a multilayer.

For complementary dGMP and dCMP base pair nucleotides, there are other effects that occur when coadsorbed onto the TiO_2_ surface. It is observed that the adsorbed spectra are highly similar to the equilibrated single-component dGMP adsorbed spectra in shape, intensity, and band positions (Figure S4c). This suggests that dGMP has a higher surface affinity than dCMP, and the surface is mainly comprised of dGMP. The adsorption kinetics are shown for the nucleobase (Figure 8b) and phosphate (Figure S5b) functional groups. The 1000 cm^–1^ ν(Ti–O–P) band shows exponential increase followed by slowing and a slight plateau, like single-component adsorption. However, for the 1489 cm^–1^ ν(C–N), δ(C–H) peak kinetics, a slight inflection point is observed around the 90 min mark. This is hypothesized to come from a change to the extinction coefficients to the 1489 cm^–1^ vibrational modes, as hydrogen-bound nucleotides can alter the dynamic dipole moment.^46−48^ After desorption, most of the nucleotides remain on the surface (Figure S6c).

There are two configurations for hydrogen-bound dGMP and dCMP complementary base pairs, specifically, Hoogsteen (HG) and Watson–Crick (WC). Previous studies report that HG is preferred at lower pH due to the protonated dCMP N3-H^+^ that hydrogen-bonds to dGMP N7 at the nitrogenous rings. WC occurs at higher pH values and is dominant at physiological pH.^48^ Additionally, for dGMP-dCMP, HG forms two H-bonds and WC forms three H-bonds. Stelling et al. investigated the interaction of duplex DNA and identified IR band assignments to either Hoogsteen or Watson–Crick hydrogen bonding conformations.^48^ When WC conformation was switched to HG, an ∼30% decrease in the 1498 cm^–1^ N7 peak intensity was noted as a spectral band marker. In our study, observation of other peak shifts as spectral makers is not seen, but it could be due to the adsorption of nucleotides onto the TiO_2_ surface. However, in Figure 8b, an ∼10% decrease in the ν(C–N), δ(C–H) is observed. The intensity of decrease is not as significant as previously reported by Stelling et al., but this could be due to the use of single nucleotides over oligonucleotides which could form multiple H-bonding configurations and cause a larger change in intensity. Additionally, at our experimental pH 5, both HG and WC could be possible, whereas, in the reference, the 30% intensity decrease is from a complete conversion from WC to HG. Therefore, a multilayer of hydrogen-bound complementary base pair nucleotides is observed to form on the surface.

At experimental pH 5, both zwitterionic and monovalent anionic forms of dCMP are present in solution; thus, the H-bonding configuration could be HG or WC. However, for the adsorbed two-component, competitive adsorption spectra, the protonated 1717 cm^–1^ ν(NH^+^) dCMP peak is not observed but is seen for adsorbed single-component. Since HG requires a protonated dCMP, the multilayer configuration must be dominated by WC; the multilayer dCMP undergoes deprotonation to adopt a WC base pairing configuration. Furthermore, WC base pairing can be more energetically stable than HG.^49^ To surmise the composition of each layer in the multilayer, previous ζ potential and the preference of pyrimidine over purine nucleotides results suggest that dGMP has a higher surface affinity and would be directly bound to the surface. The second layer could then be comprised of hydrogen-bound dCMP pyrimidine ring to the exposed purine ring of dGMP (Figure 9). This H-bonding conformation is identical to how complementary base pairs interact in DNA. The multilayer formation of complementary base pairs on metal oxide particles is evidence for biomolecular templating, providing insight into the formation of prebiotic life.

For the other complementary base pair, dAMP and dTMP (Figure S4d), it is more difficult to determine an H-bonding configuration as both HG and WC have two hydrogen bonds, and neither case requires a protonated nucleotide. However, it is possible to discern which nucleotide is preferentially directly coordinated to the surface. The nucleobase peak intensity as a function of time (Figure 8b) for the two-component system shows an inflection point for the 1480 cm^–1^ ν(C–N), δ(C–H) band at ∼90 min during adsorption. After desorption, only 20% of the 1694 cm^–1^ ν(C=O) band is seen (data not shown). This is different than the dGMP-dCMP system, where the multilayer was stabilized and only a minimal decrease in various peak intensities was observed. The 1694 cm^–1^ band can be attributed to dTMP as there is 1694 cm^–1^ present in the single-component adsorbate spectrum alone, and no such peak is present for dAMP (Figure 3c,d). The spectral shape resembles adsorbed single-component dAMP spectra than single-component dTMP. This suggests that a multilayer is formed, and the second layer reversibly adsorbs. In both WC and HG dAMP-dTMP configurations, there are only two H-bonds compared to the three H-bonds in WC dGMP-dCMP. The reversibility of the multilayer in dAMP-dTMP compared to the relative irreversibility of dGMP-dCMP could be due to the additional H-bond in WC configuration for dGMP-dCMP, leading to a more stable multilayer. For these reasons, the data show that dAMP is directly coordinated to the TiO_2_ surface while dTMP interacts within a second layer (Figure 9). There is a preferential adsorption of purine (dAMP and dGMP) over pyrimidine (dTMP and dCMP) nucleotides to TiO_2_, even in more complex systems, for the same reasons mentioned previously for single-component adsorption. Since the spectra for both complementary base pairs represent more of the adsorbed purine nucleotide, the second layer of hydrogen-bound pyrimidine nucleotides cannot be in a 1:1 ratio to the directly coordinated purine layer; the second layer does not fully cover the first adsorbed layer.

**Figure 10:**
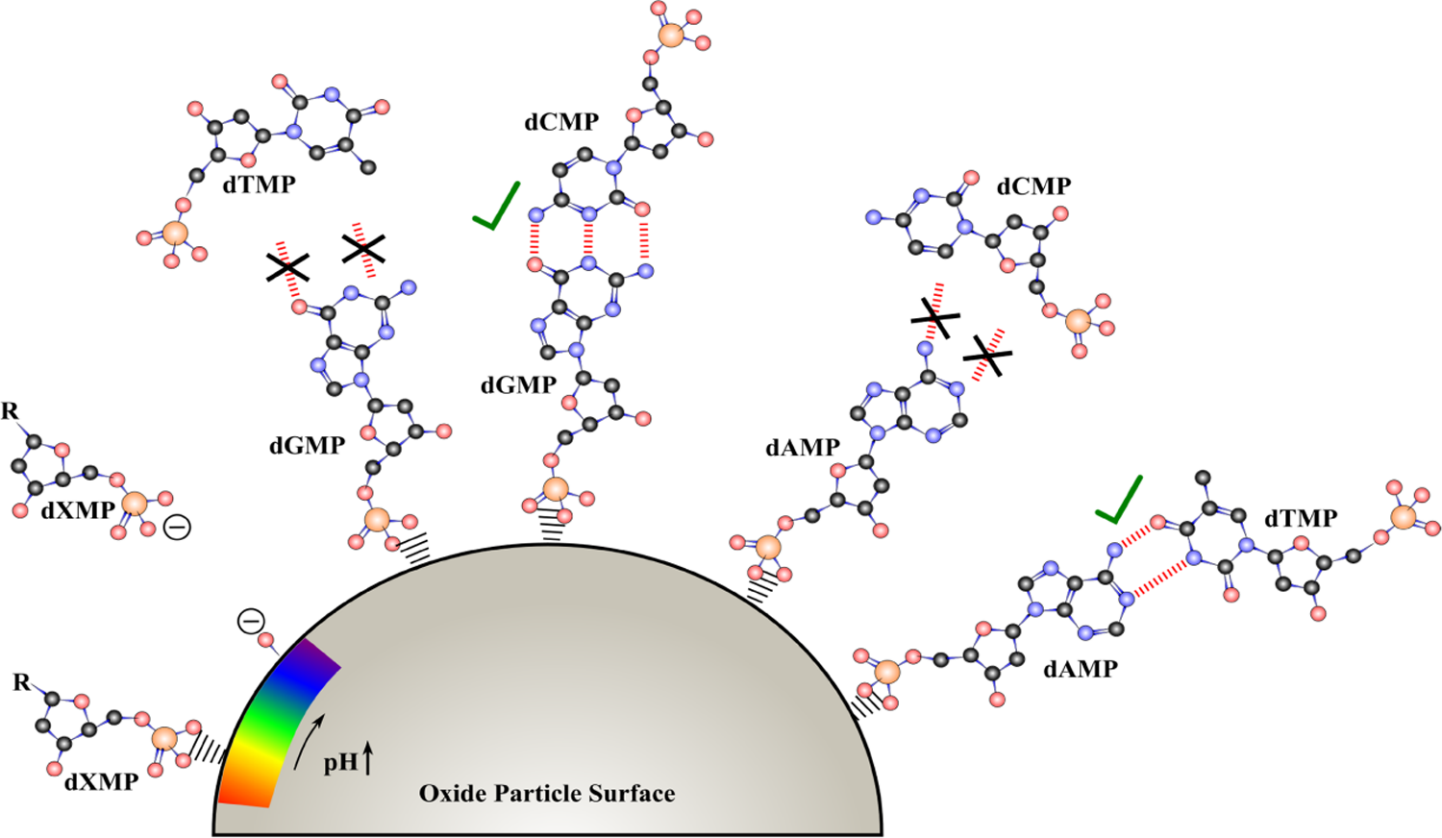
Pictorial representation of different surface chemistries occurring on an oxide particle surface for nucleotides. From left to right: electrostatic pH dependence of singly adsorbed nucleotides where nucleotides are attracted at lower pH compared to repelled at higher pH. Two-component adsorption only forms hydrogen-bound interactions between the strongly bound nucleotide layer and weaker H-bonded second layer with complementary base pairs where noncomplementary nucleotides do not form a second layer. Depicted is also the preferential adsorption of dGMP compared to dCMP and dTMP, and dAMP compared to dTMP and dCMP.

Figure 10 depicts the results of this study and shows that there are differential surface interactions and surface templating effects from pH, competitive and complementary base pair nucleotide adsorption. Additionally, this study shows the reduction of nucleotide bioavailability in solution due to adsorption on oxide particle surfaces in aqueous environments at lower pH values. Regardless of nucleotide derivative, the phosphate group is shown to directly coordinate to the oxide surface, leaving the nucleobase free to further interact with components in solution. In systems where there are multicomponent nucleotide solutions, the composition of the adsorbed layer is heavily dominated by the direct surface coordinated purine nucleotides while a second layer only forms in the presence of complementary pyrimidine nucleotides.

## Conclusions

Adsorption of biomolecular components onto geochemical mineral surfaces is important to understand as it can provide insight into the environmental DNA and surface adsorption of these components in the environment as well as the role of surfaces in the origins of prebiotic life. The results from this study show that nucleotides lead to high levels of adsorption at lower pH and little adsorption at higher pH. Spectral broadening in the phosphate band region shows how nucleotides are directly bound to the surface via the phosphate group. The binding mode appears to be similar for all nucleotides regardless of nucleobase derivative from similar phosphate band absorption shape. This suggests that the surface chemistry in this first adsorbed layer is dependent on surface composition and structure rather than a specific nucleotide composition. However, there are still differential surface interactions leading to different surface coverages and rates of adsorption. Additionally, when nucleotides are coadsorbed in a two-component system, second layer formation and specific interactions only occur for complementary base pairs but not for noncomplementary base pairs or single-component systems. Overall, the results from this study show that nucleotides can be concentrated from dilute bulk solutions onto geochemical surfaces and can template specific interactions, which has consequences for these biological components in the environment for both current considerations and in the early Earth.

## Supporting Information Available

Tabulated speciation forms for all four nucleotides at pH 5 and 9; vibrational peak assignments; ATR-FTIR spectra of adsorbed nucleotides at pH 9; phosphate absorption band region for solution and adsorbed nucleotides; single-component desorption peak height kinetics at pH 5; spectra for noncomplementary and complementary base pair adsorption; normalized phosphate peak height kinetics for two-component adsorption; and desorption peak height kinetics for two-component adsorption (PDF)
