# Correction to “Differential Surface Interactions and Surface Templating of Nucleotides (dGMP, dCMP, dAMP, and dTMP) on Oxide Particle Surfaces”

**DOI:** 10.1021/acs.langmuir.3c01397

**Published:** 2023-07-28

**Authors:** Izaac Sit, Eleanor Quirk, Eshani Hettiarachchi, Vicki H. Grassian

An incorrect version of Figure 2 was published and the correct version is provided here. The new version includes corrected pKa values along with a reference for these values.^1^ These changes have led to new speciation percentages given in the main text (page 15040 right column) from: “At pH 5 for dGMP, the amounts of the zwitterionic, monovalent anionic, and divalent anionic forms are 1.8, 91.0, and 7.2%, respectively. For dCMP at pH 5, these percentages change to 15.6, 78.2, and 6.2% for the zwitterionic, monovalent anionic, and divalent anionic forms, respectively.” to the following correct values: “At pH 5 for dGMP, the amounts of the zwitterionic, monovalent anionic, and divalent anionic forms are 0.8, 94.5, and 4.7%, respectively. For dCMP at pH 5, these percentages change to 23.1, 73.2, and 3.7% for the zwitterionic, monovalent anionic, and divalent anionic forms, respectively.”

In addition, a new table (Table S1) with speciation percentages at pH 5 and pH 9 is now provided in the updated Supporting Information (SI).
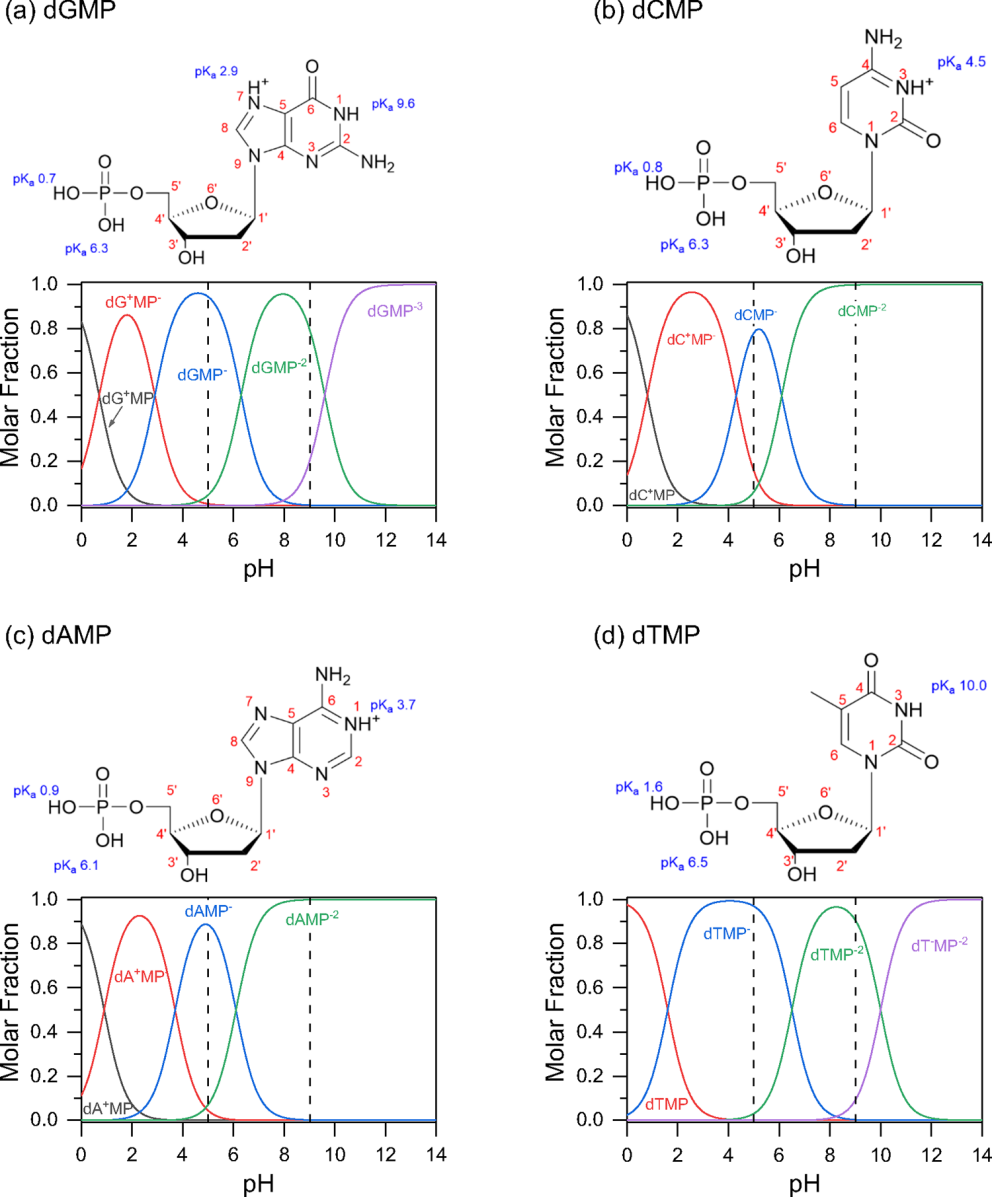


## References

[ref1] Shabarova Z. A.; Bogdanov A. A. In Advanced Organic Chemistry of Nucleic Acids; John Wiley & Sons, Ltd, 1994; pp 93–180.

