# Supplementary material for: Differential Surface Interactions and Surface Templating of Nucleotides (dGMP, dCMP, dAMP, and dTMP) on Oxide Particle Surfaces
Source: Langmuir. 2022 Nov 29;38(49):15038–49. doi: 10.1021/acs.langmuir.2c01604 (PMC9753757; doi:10.1021/acs.langmuir.2c01604)
Supplement: Supplementary file 1 — la2c01604_si_001.pdf [file la2c01604_si_001.pdf]

Supporting Information for

Differential Surface Interactions and Surface Templating of Nucleotides (dGMP, dCMP, dAMP and dTMP) on Oxide Particle Surfaces

Izaac Sit[1], Eleanor Quirk[1], Eshani Hettiarachchi[2], and Vicki H. Grassian[2]*

[1]Department of Nanoengineering and [2]Department of Chemistry and Biochemistry
University of California San Diego, La Jolla CA 92093
*Author to whom correspondence should be addressed (vhgrassian@ucsd.edu)

Table S1. Tabulated speciation forms for the dGMP, dCMP, dAMP and dTMP at pH 5 and 9 as determined using Henderson-Hasselbalch equation. Speciation forms are shown in percentages.

	**$dG^{+}MP^{-}$**	**$dGMP^{-}$**	**$dGMP^{-2}$**	**$dGMP^{-3}$**
pH 5	1.8	91.0	7.2	0.0
pH 9	0.0	0.1	66.5	33.4

	**$dC^{+}MP^{-}$**	**$dCMP^{-}$**	**$dCMP^{-2}$**	**$dCMP^{-3}$**
pH 5	15.6	78.2	6.2	0.0
pH 9	0.0	0.1	99.9	0.0

	**$dA^{+}MP^{-}$**	**$dAMP^{-}$**	**$dAMP^{-2}$**	**$dAMP^{-3}$**
pH 5	4.4	88.6	7.0	0.0
pH 9	0.0	0.1	99.9	0.0

	**dTMP**	**$dTMP^{-}$**	**$dTMP^{-2}$**	**$dTMP^{-3}$**
pH 5	0.0	96.9	3.1	0.0
pH 9	0.0	0.3	90.6	9.1

Table S2. Deoxyguanosine monophosphate and deoxycytidine monophosphate vibrational mode peak assignments in solution at pH 5 and 9 compared to adsorbed on TiO_2 nanoparticles.

Vibrational modes[a]	Vibrational Frequency (cm^{-1})								Literature Assignment (cm^{-1})
	Solution				Adsorbed				
	dGMP		dCMP		dGMP		dCMP		
	pH 5	pH 9	pH 5	pH 9	pH 5 (Δ)[b]	pH 9 (Δ)[b]	pH 5 (Δ)[b]	pH 9 (Δ)[b]	
δ($N3H^+$)			1717				1717 (+0)		1707[1], 1696[2]
ν(C6=O6)	1683	1683			1684 (+1)				1698-1678[3]
δ(HOH)	1637	1637	1649	1644	1641 (+4)	1651 (+14)	1651 (+2)	1651 (+7)	
purine/pyrmidine ring	1601		1600		1604 (+3)		1597 (+3)		1603[1], 1607[4]
purine/pyrmidine ring	1577	1577	1579	1577	1576 (-1)	1573 (-4)	1580 (+1)		1580[1], 1570[4], 1575-1590[3]
purine/pyrmidine ring	1536	1540	1529	1541	1535 (+1)		1528 (-1)		1527-1520[3]
ν(C-N), δ(C-H)	1485	1483	1491	1491	1485 (+0)		1492 (-1)	1490 (+1)	1479[1], 1495-1476[3]
purine/pyrmidine ring	1467	1467	1465	1472	1467 (+0)		1465 (+0)		1477-1785[3]
δ(CH_2)	1413	1413	1415		1412 (+1)		1416 (+1)		1422[1] ,1425[4]
purine/pyrmidine ring	1363	1363	1365	1366	1362 (-1)		1369 (+4)		1365-1360[3]
pyrmidine ring			1290	1293			1291 (+1)		1304[1], 1307[4]
ν(N9-C1')	1215				1214 (-1)				1217[1], 1212[4]
$\nu_{as}(PO_2^-)$	1175		1193		1171 (-4)		1201 (+8)		1159[5]
bidentate/monodentate					1097	1090	1095	1090	1115[5], 1105[6]
$\nu_{as}(PO_3^{2-})$		1089		1090					1084[1], 1088[4], 1078[5]
$\nu_s(PO_2^-)$	1085		1085		1070 (-15)		1070 (-15)		1077[5]
ν(P-O)	1002		1004						1002-1003[1], 996-1002[2]
ν(Ti-O-P)					1002	997	1004	994	992-997[1]
$\nu_s(PO_3^{2-})$		978		978					978-980[1], 974[4], 979[2]
δ(POH)	949	934	948	937					935[1], 935[7]
$\nu_s(P\text{-}(OTi)_2)$ of bidentate complexes					949		951		936[1], 935[8]

[a] $\nu_{s/as}$: symmetric/asymmetric stretch vibration; δ: bending vibration

[b] Δ: difference between adsorbed and solution phase wavenumber at last time point collected.

Table S3. Deoxyadenosine monophosphate and deoxythymidine monophosphate vibrational mode peak assignments in solution at pH 5 and 9 compared to adsorbed on TiO_2 nanoparticles.

Vibrational modes[a]	Vibrational Frequency (cm^{-1})								Literature Assignment (cm^{-1})
	Solution				Adsorbed				
	dAMP		dTMP		dAMP		dTMP		
	pH 5	pH 9	pH 5	pH 9	pH 5 (Δ)[b]	pH 9 (Δ)[b]	pH 5 (Δ)[b]	pH 9 (Δ)[b]	
$\delta(N1H^+)$	1710								1707[1], 1696[2]
ν(C2=O2)			1694	1694			1694 (+0)	1693 (+1)	1698-1691[3]
δ(HOH)	1651	1647	1660	1634	1647 (-4)		1661 (+1)		
purine ring	1604	1606			1605 (+1)				1603[1], 1607[4]
purine/pyrmidine ring	1579	1577		1575	1579 (+0)	1578 (+1)			1580[1], 1570[4], 1575-1590[3]
purine/pyrmidine ring		1540		1540					1527-1520[3]
ν(C-N), δ(C-H)	1480	1475	1481	1476	1480 (+0)	1477 (+2)	1481 (+0)		1479[1], 1495-1476[3]
$\delta(CH_2)$	1423	1422	1418	1418	1423 (+0)	1422 (+0)	1418 (+0)		1422[1] ,1425[4]
pyrimidine ring			1374				1373 (+0)		1389-1374[3]
purine ring	1338	1338			1339 (+1)	1338 (+0)			1344-1335[3]
purine ring	1306	1306			1305 (-1)				1304[1], 1307[4]
pyrmidine ring			1277	1278			1277 (+0)	1278 (+0)	1281-1275[3]
$\delta(C6\text{-}NH_2)$	1250	1253			1250 (+0)	1251 (-2)			1248[1], 1249[4]
ν(N9-C1')	1216	1214			1216 (+0)				1217[1], 1212[4]
$\nu_{as}(PO_2^-)$	1164		1187						1159[5]
bidentate/monodentate					1104	1101	1100	1086	1115[5], 1105[8]
$\nu_{as}(PO_3^{2-})$		1089		1086					1084[1], 1088[4], 1078[5]
$\nu_s(PO_2^-)$	1084		1081		1072 (-12)		1069 (-12)		1077[5]
ν(P-O)	1002		1007						1002-1003[1], 996-1002[2]
ν(Ti-O-P)					1003	1003	1002	999	992-997[1]
$\nu_s(PO_3^{2-})$		978		977					978-980[1], 974[4], 979[2]
δ(POH)	951	934	945	940					935[1], 935[7]
$\nu_s(P\text{-}(OTi)_2)$ of bidentate complexes					952		951		936[1], 935[8]

[a] $\nu_{s/as}$: symmetric/asymmetric stretch vibration; δ: bending vibration

[b] Δ: difference between adsorbed and solution phase wavenumber at last time point collected.

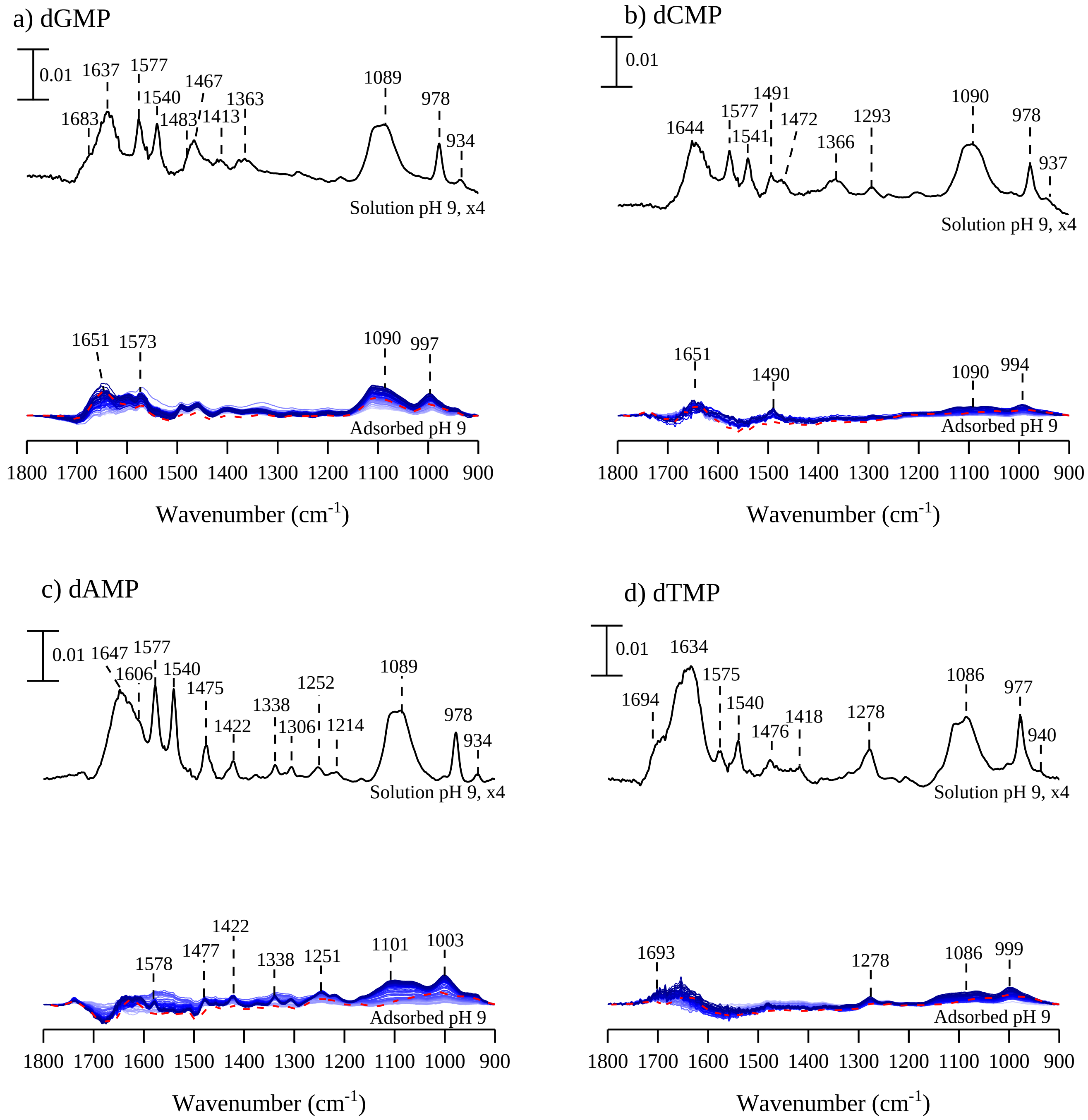

Figure S1. ATR-FTIR spectra at pH 9 of solution phase nucleotides (top) and adsorbed on TiO_2 (bottom) for a) deoxyguanosine monophosphate; b) deoxycytidine monophosphate; c) deoxyadenosine monophosphate; d) deoxythymidine monophosphate. ATR-FTIR spectra are collected as a function of adsorption time. These spectra are shown every 5 minutes from light to dark coloration. The red dotted line represents the desorption spectrum after 120 minutes. Solution phase spectra have been scaled (x4) to on the same scale bar.

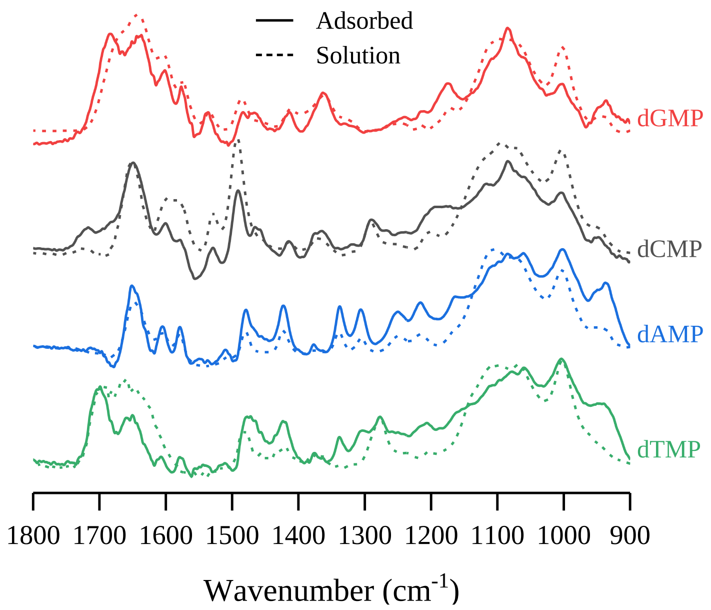

Figure S2. Overlayed normalized ATR-FTIR spectra at pH 5 for solution (dotted) and saturated adsorbed (solid) of the phosphate absorption band between 900-1200 cm^{-1} for dGMP, dCMP, dAMP, and dTMP.

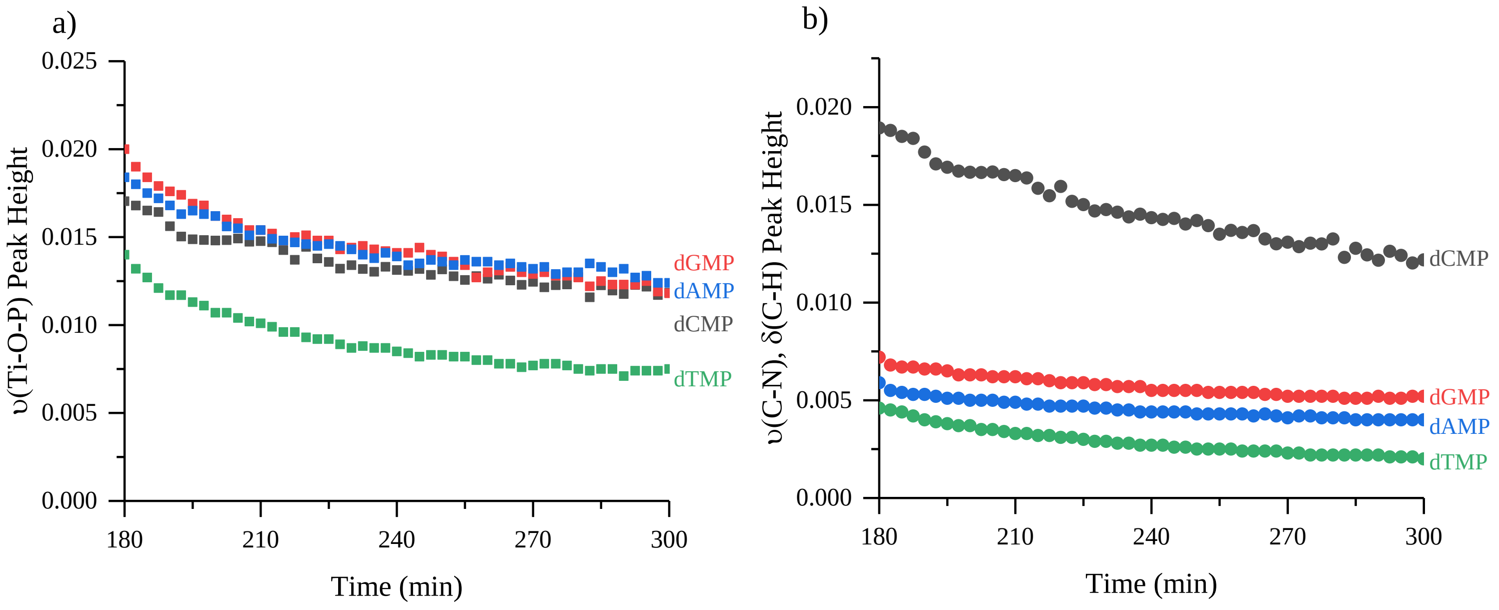

Figure S3. One component nucleotide adsorption peak height desorption kinetics onto TiO_2 at pH 5 using the (a) ~1000 cm^{-1} ν(Ti-O-P) peak height and (b) ~1490 cm^{-1} ν(C-N), δ(C-H) vibrational bands.

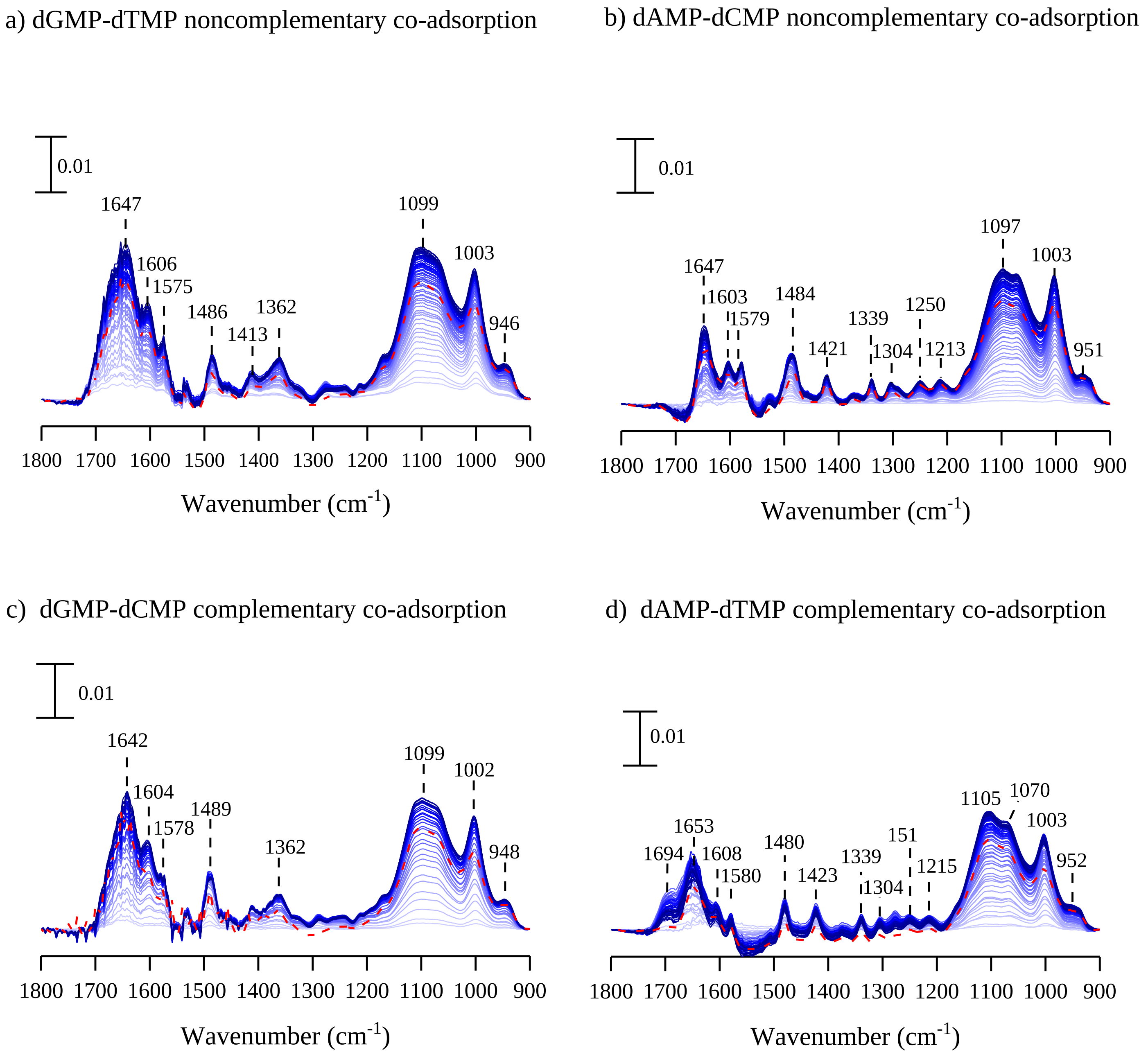

Figure S4. ATR-FTIR spectra at pH 5 of equimolar base pair solution adsorbed on TiO_2 for a) dGMP-dTMP noncomplementary, b) dAMP-dCMP noncomplementary, c) dGMP-dCMP complementary and d) dAMP-dTMP complementary. ATR-FTIR spectra are collected as a function of adsorption time. These spectra are shown every 5 minutes from light to dark coloration. The red dotted line represents the desorption spectrum after 120 minutes.

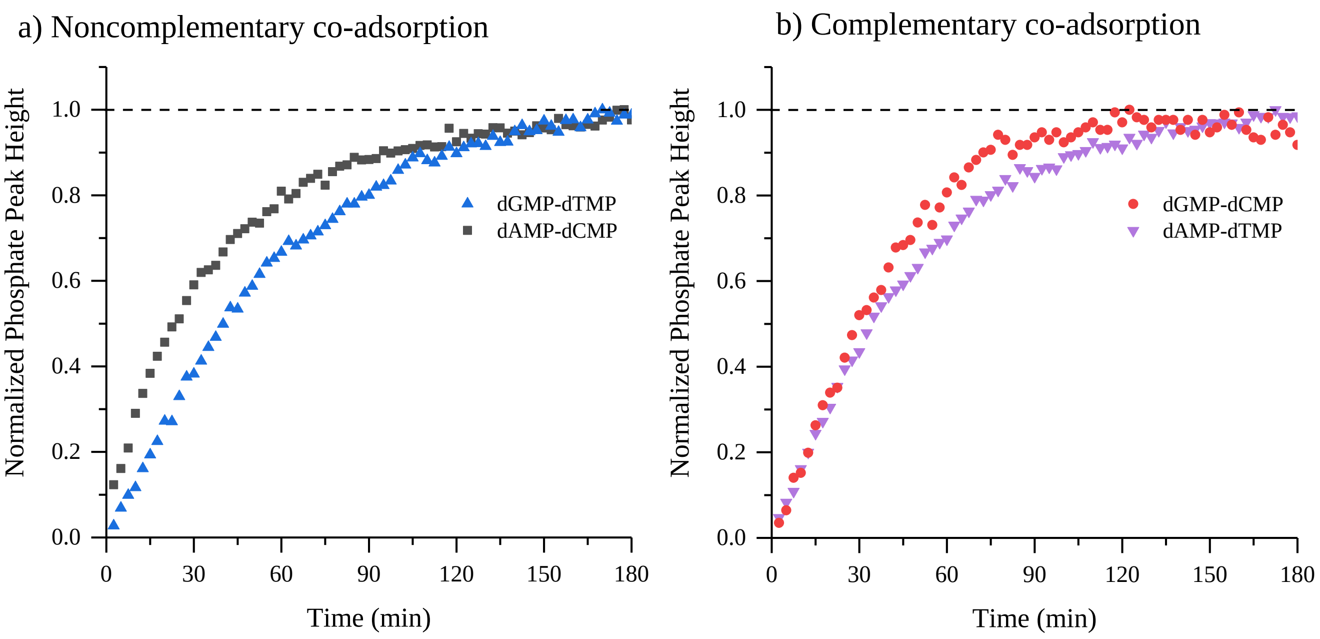

Figure S5. Changes to normalized phosphate peak height for two component nucleotide base pair adsorption onto TiO_2 at pH 5 using the ca. 1000 cm^{-1} ν(Ti-O-P). a) noncomplementary nucleotide base pairs dGMP-dTMP (blue triangle) and dAMP-dCMP (gray square). b) complementary nucleotide base pairs dGMP-dCMP (red circle) and dAMP-dTMP (purple upside-down triangle).

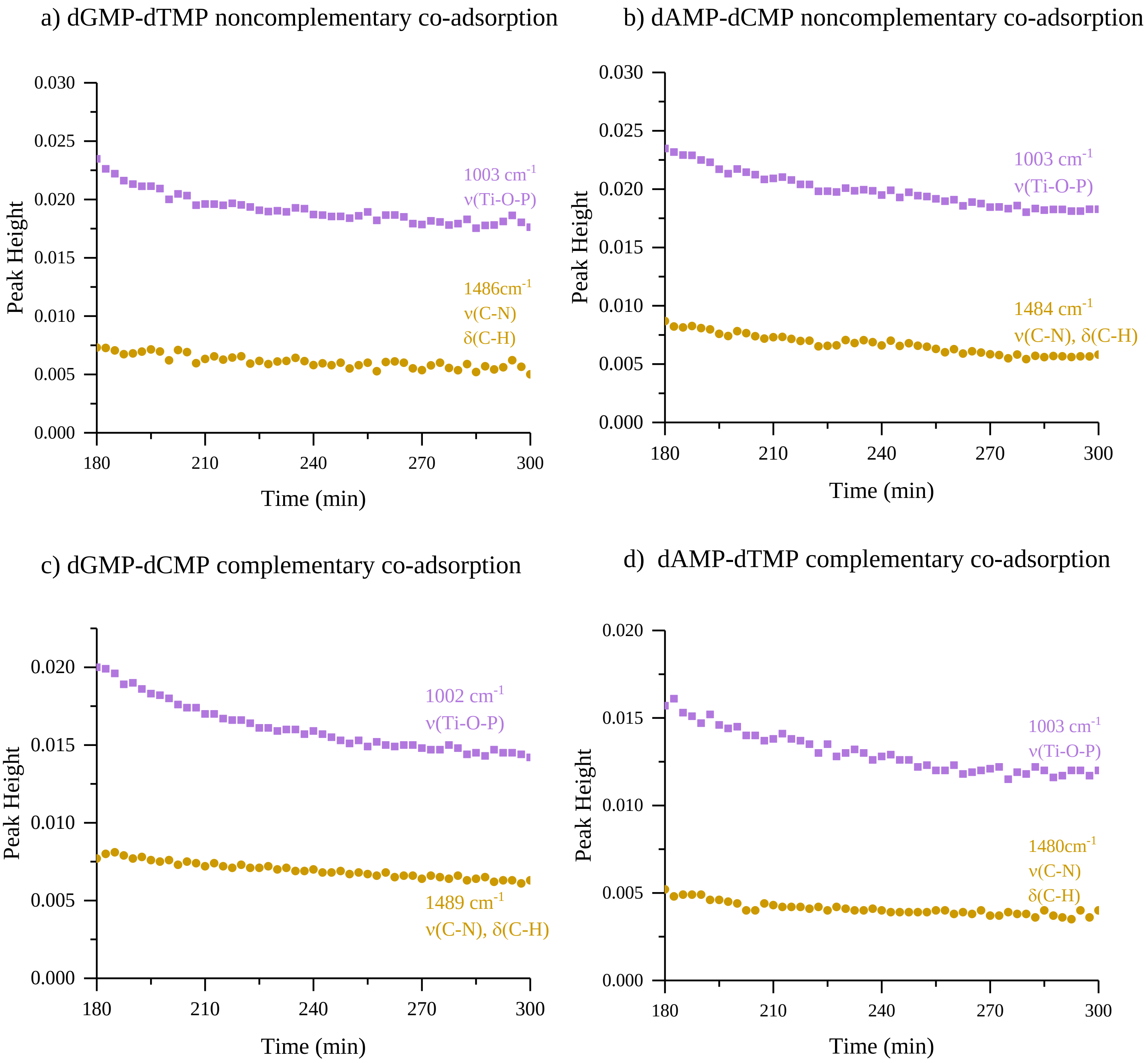

Figure S6. Changes to nucleotide peak height desorption of the ca. 1003 cm^{-1} ν(Ti-O-P) (purple) and ca. 1486 cm^{-1} ν(C-N), δ(C-H) (gold) onto TiO_2 at pH 5 for two component a) dGMP-dTMP noncomplementary, b) dAMP-dCMP noncomplementary, c) dGMP-dCMP complementary, and d) dAMP-dTMP complementary base pairs.

References

(1) Sit, I.; Sagisaka, S.; Grassian, V. H. Nucleotide Adsorption on Iron(III) Oxide Nanoparticle Surfaces: Insights into Nano–Geo–Bio Interactions Through Vibrational Spectroscopy. *Langmuir* **2020**, *36* (51), 15501–15513. https://doi.org/10.1021/acs.langmuir.0c02633.

(2) Parikh, S. J.; Chorover, J. ATR-FTIR Spectroscopy Reveals Bond Formation During Bacterial Adhesion to Iron Oxide. *Langmuir* **2006**, *22* (20), 8492–8500. https://doi.org/10.1021/la061359p.

(3) Banyay, M.; Sarkar, M.; Gräslund, A. A Library of IR Bands of Nucleic Acids in Solution. *Biophys. Chem.* **2003**, *104* (2), 477–488. https://doi.org/10.1016/S0301-4622(03)00035-8.

(4) Tajmir-Riahi, H.-A.; Messaoudi, S. The Effects of Monovalent Cations Li+, Na+, K+, NH4 +, Rb+ and Cs+ on the Solid and Solution Structures of the Nucleic Acid Components. Metal Ion Binding and Sugar Conformation. *J. Biomol. Struct. Dyn.* **1992**, *10* (2), 345–365. https://doi.org/10.1080/07391102.1992.10508652.

(5) Elzinga, E. J.; Sparks, D. L. Phosphate Adsorption onto Hematite: An in Situ ATR-FTIR Investigation of the Effects of pH and Loading Level on the Mode of Phosphate Surface Complexation. *J. Colloid Interface Sci.* **2007**, *308* (1), 53–70. https://doi.org/10.1016/J.JCIS.2006.12.061.

(6) Connor, P. A.; McQuillan, A. J. Phosphate Adsorption onto TiO_2 from Aqueous Solutions: An in Situ Internal Reflection Infrared Spectroscopic Study. *Langmuir* **1999**, *15* (8), 2916–2921. https://doi.org/10.1021/la980894p.

(7) Wu, R. R.; He, C. C.; Hamlow, L. A.; Nei, Y. -w.; Berden, G.; Oomens, J.; Rodgers, M. T. N3 Protonation Induces Base Rotation of 2′-Deoxyadenosine-5′-Monophosphate and Adenosine-5′-Monophosphate. *J. Phys. Chem. B* **2016**, *120* (20), 4616–4624. https://doi.org/10.1021/acs.jpcb.6b04052.

(8) Yan, W.; Jing, C. Molecular Insights into Glyphosate Adsorption to Goethite Gained from ATR-FTIR, Two-Dimensional Correlation Spectroscopy, and DFT Study. *Environ. Sci. Technol.* **2018**, *52* (4), 1946–1953. https://doi.org/10.1021/acs.est.7b05643.
